# Endoscopic Retroperitoneal Adrenalectomy for Adrenal Metastases

**DOI:** 10.1155/2014/806194

**Published:** 2014-09-08

**Authors:** Gintaras Simutis, Givi Lengvenis, Virgilijus Beiša, Kęstutis Strupas

**Affiliations:** ^1^Clinic of Gastroenterology, Nephrourology and Surgery, Center of Abdominal Surgery, Faculty of Medicine, Vilnius University, Santariškiu 2, 08661 Vilnius, Lithuania; ^2^Faculty of Medicine, Vilnius University, M.K.Čiulionio 21, 03101 Vilnius, Lithuania

## Abstract

*Objectives*. To evaluate whether retroperitoneal approach for adrenalectomy is a safe and effective treatment for adrenal metastases (AM). *Methods*. From June 2004 to January 2014, nine consecutive patients with AM were treated with endoscopic retroperitoneal adrenalectomy (ERA). A retrospective study was conducted, and clinical data, tumor characteristics, and oncologic outcomes were acquired and analyzed. *Results*. Renal cancer was the primary site of malignancy in 44.4% of cases. The mean operative time was 132 ± 10.4 min. There were 5 synchronous and 4 metachronous AM. One patient required conversion to transperitoneal laparoscopic procedure. No mortality or perioperative complications were observed. The median overall survival was 11 months (range: 2–42 months). Survival rates of 50% and 25% were identified at 1 and 3 years, respectively. At the end of the study, 4 patients were alive with a mean observed follow-up of 20 months. No patients presented with local tumor relapse or port-site metastases. *Conclusions*. This study shows that ERA is a safe and effective procedure for resection of AM and advances the surgical treatment of adrenal disease. The use of the retroperitoneal approach for adrenal tumors less than 6 cm can provide very favorable surgical outcomes.

## 1. Introduction

Metastases to the adrenal glands represent the second most common type of adrenal mass after adrenal adenomas [1]. Lung, breast, stomach, and kidney cancers and melanomas and lymphomas most commonly metastasize to the adrenal glands [2]. Management strategy of adrenal metastases (AM) varies depending on the different clinical situation and can include close observation, chemotherapy, local ablative therapy, radiotherapy, or surgical resection [3–6]. Several studies have confirmed prolonged survival after an adrenal metastasectomy in selected patients who presented with isolated AM when that is the only site of disease spread [7–10].

Over the last few decades, laparoscopic technique radically changed adrenal surgery, making access to the adrenal glands easier and less traumatic. Laparoscopic adrenalectomy (LA) has become the gold standard for removing benign tumors of the adrenal glands because it offers lower morbidity rates, reduced postoperative pain, shorter hospital stay, perfect cosmetic results, and other benefits compared to open surgery [11, 12]. LA could be performed transperitoneally or retroperitoneally [13, 14]. The advantages of the transperitoneal approach include the wider working space and readily identifiable anatomic landmarks [14]. The retroperitoneal approach was considered to be associated with more direct access to the gland, avoidance of intraperitoneal organs, avoidance of adhesions in previously operated patients, and the ability to perform bilateral adrenalectomy without repositioning [15]. Different surgical methods could be selected according to the characteristics of patients, such as tumor diameter, location, histologic type, extent of deterioration, and metastasis [16].

Advantages of laparoscopic surgery have prompted interest in expanding this method to the treatment of adrenal malignancies [17–19]. Long-term survival after LA for isolated AM was demonstrated in several reports [3, 7]; however, the utility of laparoscopic methods for malignancies is less certain because of concerns regarding the risk of tumor cell spillage [20].

Although controversial, LA was recommended as an appropriate initial approach for isolated AM in some studies, because it achieved the same level of results of tumor control and less traumas compared with open surgery [21–23]. Furthermore, there was no quantitative assessment concerning the association between retroperitoneal adrenalectomy and patients with AM. In response, we conducted the study to evaluate the efficacy and effectiveness of endoscopic retroperitoneal adrenalectomy (ERA) for AM.

## 2. Materials and Methods

From June 2004 to January 2014, 145 ERA were performed at Vilnius University Hospital Santariskiu Klinikos. Nine patients (6,25%) were found to have a histologically confirmed AM. These 9 patients were included in the present retrospective study.

The diagnosis of AM from primary tumor was suspected in any case of newly diagnosed adrenal mass, characterized by growing size on sequential imaging studies (abdominal ultrasonosgraphy and computed tomography (CT)). Percutaneous adrenal biopsy was ruled out for prevention of tumor seeding. All patients were evaluated with the assistance of an oncologist, with tumor-specific markers and global imaging (CT of the chest, abdomen, and pelvis) to search for other sites of metastatic involvement. The hormonal examination for adrenal metabolic dysfunction was carried out in conjunction with endocrinologist.

ERA was decided for patients with solitary adrenal mass inferior to 6 cm without evidence of periadrenal malignant infiltration or regional lymphadenopathy on imaging examinations, negative serologic tests for adrenal metabolic dysfunction, and patient history of malignant disease. Surgery was performed with curative intent in all patients.

Patient demographic characteristics (age, gender, tumor size and side, diagnosis of primary malignancy, and operative history) are summarized in Table 1.

Operation reports were reviewed to obtain operative time, estimated blood loss, need for conversion to transperitoneal LA or open adrenalectomy, status of resection margin, and complications. In addition, information regarding postoperative course (postoperative hospital stay, postoperative complications, the use of adjuvant therapy within 1 year of the procedure, and survival rates) was recorded. Pathology reports were reviewed to obtain removed tumor weight and final diagnosis.

Metastases were considered as synchronous (<6 months) or metachronous (≥6 months) depending on the interval after primary surgery. The completeness of adrenal surgery was defined in terms of R0 (complete resection with no microscopic residual tumor), R1 (complete resection with no grossly visible tumor as defined by the surgeon, but margins are microscopically positive according to the pathologist), R2 (partial resection, with grossly visible tumor left behind), and RX (presence of residual tumor cannot be assessed). Local recurrence was defined as radiological or biopsied confirmation of a recurrent disease in the adrenal bed. After surgery, the patients were followed up by endocrinologist and an oncologist every 6 months by physical examination and systemic CT, or sooner if they become symptomatic. Overall survival was calculated from the time of adrenalectomy up to death or end of the follow-up.

### 2.1. Surgical Technique

The posterior retroperitoneal approach was used for all ERA as described by Walz et al. [24]. After induction of general anesthesia, the patient is placed prone, in the jackknife position. A 2 cm sized transverse skin incision is made just below the tip of the 12th rib. The abdominal wall of the back is then opened and the retroperitoneal space exposed. A small cavity in the retroperitoneum is then prepared using a finger to accommodate the other trocars. A 10 mm trocar is then inserted through as second incision (4 cm removed medially from the first incision) guided by an index finger inserted through the first incision. A third skin incision for another 10 mm trocar is made along with the lowest margin of the 11th rib 4 cm laterally from the first incision. A 10 mm blunt trocar is inserted through the first incision, and carbon dioxide is insufflated to 20 mm Hg for creation of capnoretroperitoneum. The skin sutures were secured around the gas port preventing a gas leak. After creating the retroperitoneal working space, Gerota's fascia is opened, perirenal fat is dissected, and the kidney upper pole is mobilized to expose the adrenal gland. Dissection of gland starts with lower margin detachment from the upper kidney pole in a lateral to medial direction using 5 mm ultrasonic dissector. After exposing adrenal gland from surrounding tissue and medial isolation of the main suprarenal vein, the vessel is clipped and divided with scissor. Adrenal gland with surrounding fat was resected with the greatest of care to prevent tumor disruption. The surgical specimens were always extracted in a bag and retrieved via the first trocar site. No drain was inserted and the incision was closed subcutaneously.

In a case when ERA cannot safely be performed we convert it to transperitoneal LA. It is necessary to change the patient's position on the operating table. Patient is placed in the lateral decubitus position. The surgical technique for transperitoneal LA has previously been described by our team in detail [25, 26].

### 2.2. Statistical Analysis

Categorical variables are expressed as frequencies and percentages and continuous variables as mean and standard deviation (±SD). Follow-up time variable and survival time are expressed as median values. Survival probabilities were estimated by using the Kaplan-Meier method. The level of statistical significance was set at *P* < 0.05. We conducted all statistical analyses using SPSS version 13.0 for Windows (SPSS Inc.).

## 3. Results

Patients' characteristics are summarized in Table 1. The mean age of all patients undergoing ERA for AM was 66.4 ± 3.6 years. The female/male ratio was 1/1.25. All AM were unilateral (left: five patients; right: four patients). The primary sources of the metastasis are shown in Table 1. The most common primary tumor site was kidney (4 patients, 44%), followed by colon (2 patients, 22%). All patients have been operated on for primary malignancy previously. The surgical procedure consisted of contralateral ERA in all patients with previous kidney cancer. The mean tumor size in the preoperative radiologic imaging studies was 37 ± 4.5 mm. Five (56%) patients presented with synchronous metastases, while four (44%) patients presented with metachronous metastases. No percutaneous adrenal biopsy was performed for the diagnosis of malignancy.

Perioperative and postoperative data are presented in Table 2. The mean operative time and estimated blood loss during ERA were 132 ± 10.4 min and 31 ± 7 mL, respectively. One conversion to transperitoneal LA occurred because of dense adhesions and difficult interpretation of anatomical structures of retroperitoneal space caused by previous left colon resection. After open insertion of the first optical trocar in to the abdominal cavity we found few intra-abdominal adhesions that do not interfere successfully to perform LA. The operation time was 120 min in this case. There were no perioperative complications and mortality. The mean postoperative hospital stay was 3.7 ± 0.7 days.

Five patients (55,5%) had complete macroscopic resection and negative margins (R0 resection). Four patients (44,5%) had complete macroscopic resection too but margins on pathology examination were not assessed because the surgical specimen was morcellated with forceps in a bag to make it easier to remove via trocar incision (RX resection).

In all cases, pathological examinations revealed the diagnosis of metastasis related to primary malignancies. The mean specimen weight in the pathologic studies was 45.9 ± 11.4 g.

**Figure 1:**
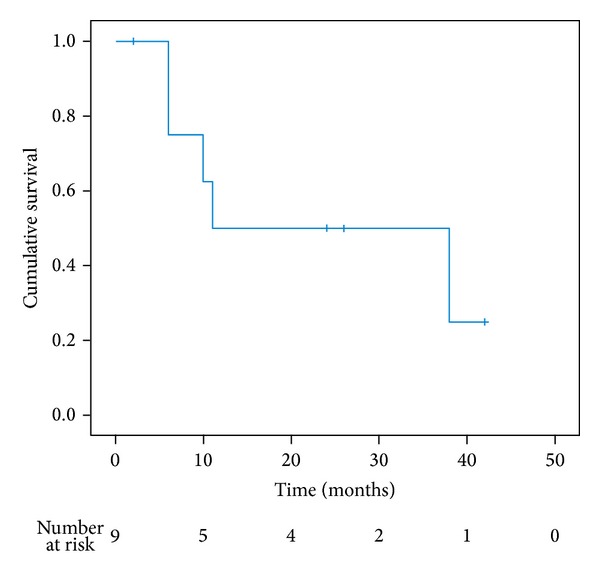
Overall survival curve according to the Kaplan-Meyer method.

The mean observed follow-up (FU) was 20 (range: 2–42) months. The median overall survival was 11 months (range: 2–42 months, Figure 1). Survival at 1 year was 50% (95% CI, 15–77; Figure 1) and at 3 years, 25% (95% CI, 1–64).

**Figure 2:**
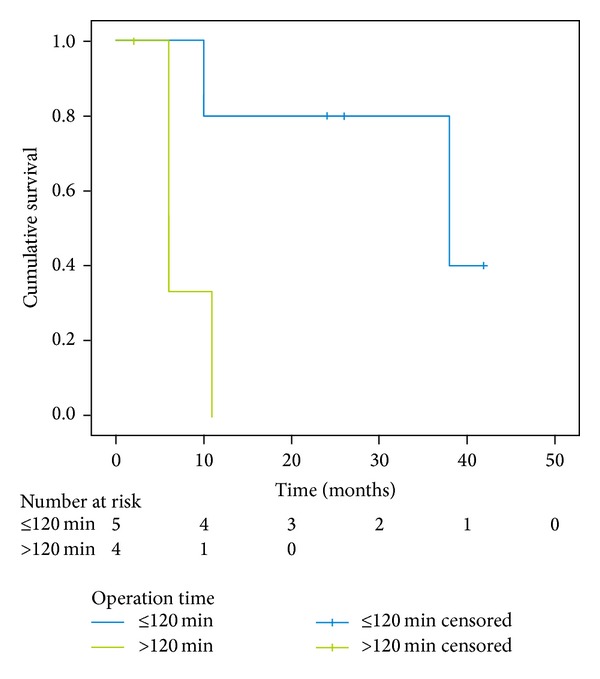
Kaplan-Meier survival curve of subgroups of patients with standard duration (≤120 min) and long duration (>120 min) of surgery; *P* = 0,024.

The difference between the median survival for patients with standard duration and long duration of surgery was significant (*P* = 0,024) (Figure 2). In patients with standard duration of surgery (≤120 min) median survival was 38 months (95% CI, 23, 25–44, 75) compared with long duration of surgery (>120 min) where median survival was 6 months (95% CI, 4, 4–10, 93).

**Figure 3:**
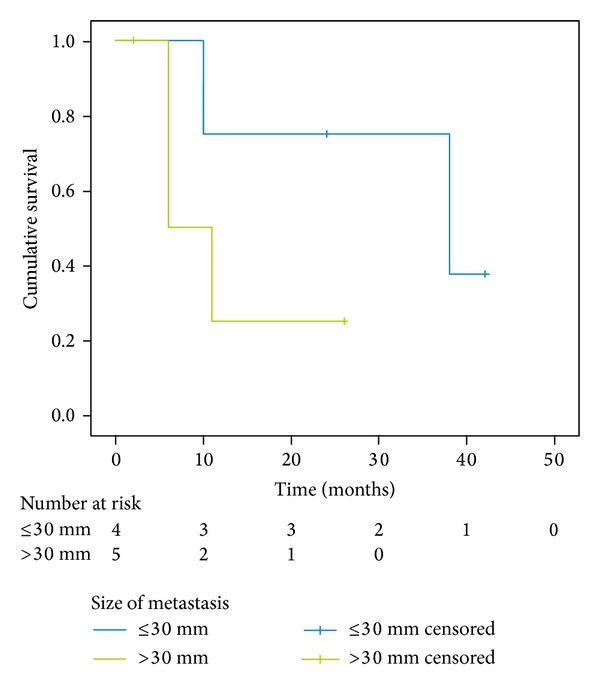
Kaplan-Meier survival curve of subgroups of patients with small (≤3 cm) and big (>3 cm) tumors; *P* = 0,180.

**Figure 4:**
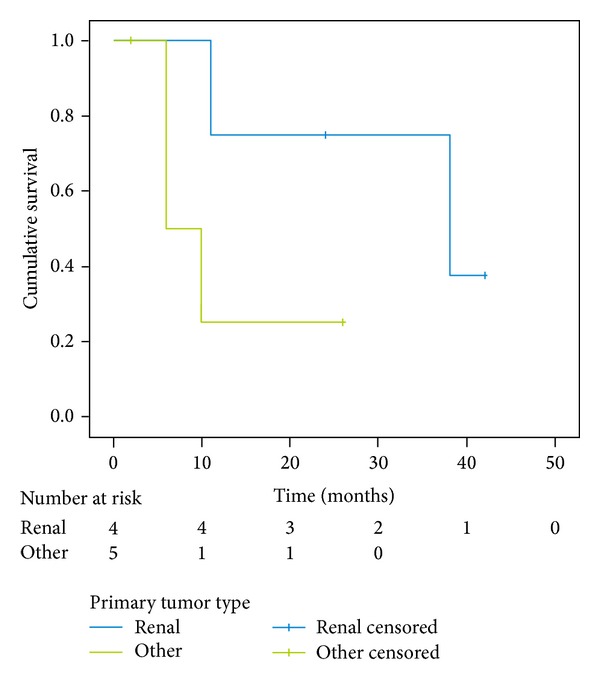
Kaplan-Meier survival curve of subgroups of patients with different origin of metastases (kidney versus other); *P* = 0,103.

**Figure 5:**
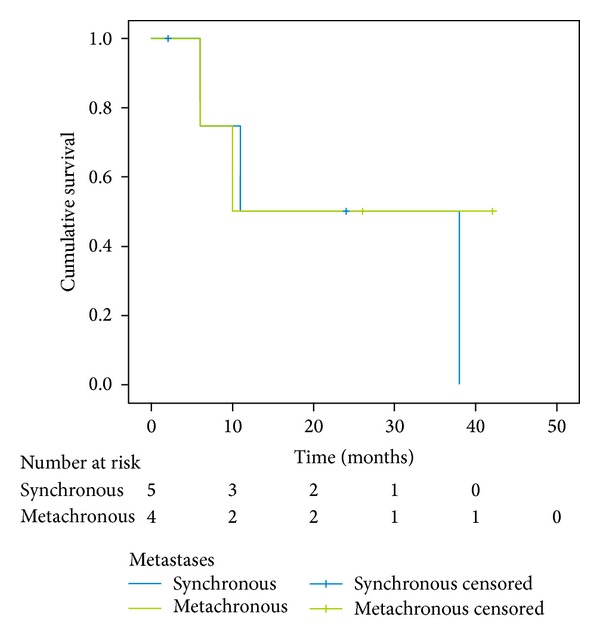
Kaplan-Meier survival curve of subgroups of synchronous and metachronous metastases; *P* = 0,711.

The differences between the median survival for patients with small and big tumors (*P* = 0,180; Figure 3), with different origin of metastases (*P* = 0,103; Figure 4) and with synchronous and metachronous metastases (*P* = 0,711; Figure 5), were not significant.

At the end of the study, 4 (45%) patients were alive with a mean FU of 20 months. Three patients were alive without evidence of disease 42, 26, and 24 months after ERA. The longest survival (42 months) was observed in a female patient with metachronous AM and primary malignancy of kidney. Causes of death in the 4 patients who did not survive 1 year were progression of disease in 3 (75%) and causes unrelated to the malignant disease in 1 (25%). No local tumor recurrence or port-site metastases were observed during follow-up period.

## 4. Discussion

The role of minimally invasive surgery for adrenal malignancies remains controversial. The number of patients successfully undergoing LA reported by most authors is small, and many studies have failed to stratify patients according to whether they had primary adrenal cancer or metastatic disease [9, 18, 19, 27]. These two conditions must be assessed separately [28]. Metastasis to the adrenal gland should be suspected in patients with adrenal incidentaloma and a history of cancers most frequently metastasizing to the lung, breast, kidney, or colon [28]. Many reports in the literature have demonstrated success of LA in cases with solitary metastases, achieving a very low incidence of local recurrences or peritoneal dissemination [17, 29, 30]. The main aspect to consider for the success of laparoscopy is a small size of the adrenal tumor. In present study, the mean diameter of such metastases was 3.7 cm and we performed ERA in all cases.

The main concern of the surgical procedure is to make the patient tumor-free and it is therefore of a great importance to follow oncological principles [30]. We recommend excision of any adrenal metastases without touching the tumor or the gland; the surgeon should start the procedure from the perirenal fat tissue, to avoid the risk of tumor spillage or incomplete resection. Retroperitoneal approach is the ideal way in order to fulfill these objectives.

We evaluated the safety and efficiency of ERA for AM and found a trend toward decreased operation time (132 min versus 144 min), blood loss (31 mL versus 130 mL), and complication rate (0% versus 8,35%) in our study group compared to results of LA for metastasis in other published series summarized in Table 3 [7, 20, 23, 28, 30–38].

Walz et al. [24] reported that despite the narrower working space and unfamiliar retroperitoneal landmarks, the ERA was associated with decreased operative time and rapid patient recovery. The local recurrence rate was satisfied, which may be attributed to the en bloc resection of metastases in ERA. The mean postoperative hospital stay 3,7 days was similar like that in other studies [9, 22, 23]. Approving that ERA for AM is superior to LA based on our data is still too early due to lack of a control group and low number of cases in our series.

Despite potential improved surgical outcomes, the oncological outcomes of laparoscopic approach have remained in question with concerns about inadequate oncological resection. In the largest series to date of patients undergoing adrenal metastasectomy, Strong et al. [23] reported a 5-year survival of 31% in 92 patients. When they compared the survival rate between the open and laparoscopic groups, they found no differences, suggesting equal oncological outcomes between the two approaches. Moreno et al. [39] also reported that the median overall survival was 29 months and 5-year survival rate was 35% after adrenalectomy for solid tumor. In our study, the median overall survival was 11 months and 1 and 3 years survival rates were 50% and 25%. The longest survival was found in patients with metastases for renal carcinoma and colorectal cancer. Even in situations of single metastasis, the survival rate was less than two years and only three patients were in complete remission of their disease. One reason for these differences may come from the populations studied.

In the literature, the prognostic factors for survival after resection for adrenal metastasis were variable, including tumor type, tumor size, operation occasion (synchronous or metachronous), margin status, and previous surgery for metastases. Ma et al. [40] found that body mass index (BMI), tumor type, tumor size, and margin status were four independent prognostic factors of survival. Renehan et al. [41] concluded that increased BMI is associated with increased risk of common and less common malignancies.

Tumor type was previously suggested to be an important prognostic factor for survival. Lo et al. [8] suggested that patients with metastases from adenocarcinoma had the best chances of survival. However, in our material, kidney cancer had the best survival period, whereas patients with melanoma and colon cancer had the worst survival. The difference in survival period may be attributed to the intrinsic biological behavior of different tumor types or presence of occult concurrent metastases in other organs. Therefore all patients have to carefully undergo preoperative staging including chest, abdominal, and cerebral CT scans before the operation.

Tumor size also was a predictive factor in previous studies. Strong et al. [23] reported that the <4.5 cm group had a better survival period than the ≥4.5 cm group. In our study, the median survival period of the ≤3 cm group was 38 versus 6 months for the >3 cm group. The difference may be attributed to the increased size of adrenal lesions correlated with the operative complexity and possible subsequent disease behavior. We also found that standard duration of surgery is statistically significant associated with better survival rate (*P* = 0,024), but these findings are limited by the low number of cases in our series.

Metachronous metastases had better survival rate in several studies [34, 42]. This result can be attributed to the different intrinsic biology of the tumors in the two groups (metachronous versus synchronous). Synchronous lesions were more aggressive and grew faster than metachronous ones. In the current study, the median survival period of the metachronous group was 10 versus 11 months for the synchronous group. The difference between the median survival for the patients with synchronous and metachronous metastases was not significant in our material; thus we cannot consider the time between findings of the primary tumor and the metastasis in adrenal gland as a strong survival predictor.

In conclusion, many studies have documented that surgery for AM contributes to a more favorable prognosis than when these tumors are not resected [3, 30]. In agreement with Zografos et al. [43] our study shows that the retroperitoneal approach can be justified and is feasible for adrenal metastases less than 6 cm. Minimally invasive surgery gives us an opportunity to minimize surgical trauma which also may be more tolerable to a patient with several previous surgical procedures.

In summary, we found that ERA for AM offers the same advantages as those amply reported for benign adrenal disease, with no morbidity and mortality, and the acceptable oncological results.

## Figures and Tables

**Table 1:** Patient characteristics and perioperative data.

Patient	Age (years)	Gender	Location	Primary malignancy	Metastases type	Treatment for primary malignancy
1	69	M	Right	Kidney cancer	S	Surgery, chemotherapy
2	65	F	Left	Lung cancer	M	Surgery, chemotherapy
3	46	M	Right	Melanoma	M	Surgery, chemotherapy
4	70	F	Left	Kidney cancer	M	Surgery
5	80	M	Right	Colon cancer	S	Surgery, chemotherapy
6	81	F	Right	Colon cancer	M	Surgery, chemotherapy
7	60	M	Left	Kidney cancer	S	Surgery
8	68	F	Left	Kidney cancer	S	Surgery
9	59	M	Left	Stomach cancer	S	Surgery, chemotherapy

F: female; M: male; S: synchronous; and M: metachronous.

**Table 2:** Perioperative and postoperative data.

Patient	Tumor size (mm)	Operative time (min)	Blood loss (mL)	Resection status	Specimen weight (g)	Hospital stay (days)	Survival (months)
1	30	110	50	R0	13	1	38
2	25	120	10	R0	15	5	10
3	40	135	10	RX	40	5	6
4	30	90	10	R0	46	2	42∗
5	60	195	50	RX	64	5	6
6	40	120	20	RX	88	8	26∗
7	15	120	20	R0	9	3	24∗
8	43	140	60	R0	32	3	11
9	50	165	50	RX	106	2	2∗

*Still alive.

**Table 3:** Surgical results of LA for metastasis in previously published series.

Author	Year	Number of patients	OT (min)	EBL (mL)	Conversion rate (%)	Complication rate (%)
Heniford et al. [7]	1999	8	181	138	10	9
Valeri et al. [31]	2001	8	160	260	0	0
Sarela et al. [34]	2003	11	NR	NR	0	NR
Sebag et al. [32]	2006	16	NR	NR	31	18,7
Castillo et al. [33]	2007	32	87	89	0	6
Adler et al. [20]	2007	9	165	63	11	0
Strong et al. [23]	2007	31	175	106	NR	NR
Marangos et al. [30]	2009	31	104	100	3,2	7,4
Crenn et al. [35]	2011	13	174	351	23	NR
Zakoji et al. [36]	2012	5	142	38	NR	NR
Toniato [28]	2013	15	80	NR	6,7	6,7
Chen et al. [37]	2014	21	159	NR	14	19
Hirayama et al. [38]	2014	8	156	30	NR	NR

Total		195	144	130	9,9	8,35

OT means operation time, EBL means estimated blood loss, and NR means not reported.
